# Conspiracy theory and cognitive style: a worldview

**DOI:** 10.3389/fpsyg.2015.00206

**Published:** 2015-02-25

**Authors:** Neil Dagnall, Kenneth Drinkwater, Andrew Parker, Andrew Denovan, Megan Parton

**Affiliations:** Department of Psychology, Manchester Metropolitan University, ManchesterUK

**Keywords:** schizotypy, delusional ideation, conspiracist belief, worldview, cognitive style

## Abstract

This paper assessed whether belief in conspiracy theories was associated with a particularly cognitive style (worldview). The sample comprised 223 volunteers recruited via convenience sampling and included undergraduates, postgraduates, university employees, and alumni. Respondents completed measures assessing a range of cognitive-perceptual factors (schizotypy, delusional ideation, and hallucination proneness) and conspiratorial beliefs (general attitudes toward conspiracist thinking and endorsement of individual conspiracies). Positive symptoms of schizotypy, particularly the cognitive-perceptual factor, correlated positively with conspiracist beliefs. The best predictor of belief in conspiracies was delusional ideation. Consistent with the notion of a coherent conspiratorial mindset, scores across conspiracy measures correlated strongly. Whilst findings supported the view that belief in conspiracies, within the sub-clinical population, was associated with a delusional thinking style, cognitive-perceptual factors in combination accounted for only 32% of the variance.

## INTRODUCTION

The study of conspiracism is important for myriad reasons. Conspiracy theories persist within the general population (Goertzel, 1994), are frequently endorsed, and may influence perceptions of significant contemporary (e.g., transmission of ebola) and historical real world events (e.g., moon landings; Swami et al., 2010). Illustratively, Stempel et al. (2007) found that 36% of American respondents believed it was at least somewhat likely that their government assisted, or took no action to stop the 9/11 attacks. Soni (2007) reported comparable results in Britain following the 7/7 bombings. Correspondingly, almost a third of the American population believed that Barack Obama unconstitutionally ascended to the US presidency (Uscinski et al., 2011). Whilst, conspiracy theories are not always false (e.g., Watergate), they typically lack evidential support and are generally resistant to falsification (Sutton and Douglas, 2014). Thus, the fact that conspiracies gain social support demonstrates the appealing, persuasive, and plausible nature of conspiracist ideas (Byford and Billig, 2001). Belief in conspiracies may also effect behavior and have important social consequences, such as diminished social engagement (society, politics, health behaviors, climate change, etc.; Butler et al., 1995; Bird and Bogart, 2005; Jolley and Douglas, 2014).

Despite a wealth of research, there is no one single, accepted definition of conspiracy (Sunstein and Vermeule, 2009). Quintessentially, theories involve the assumption of collusion, when other elucidations are more credible (Aaronovitch, 2009). This typically, manifests as the belief that multiple actors cooperate in order to orchestrate a malevolent plot (Barron et al., 2014). Conspiracy endorsement occurs, when either no definitive explanation for an event exists, or the official account appears inadequate (Drinkwater et al., 2012).

The purpose of the present paper was to extend recent work examining relationships between cognitive-perceptual variables personality factors (schizotypy: Darwin et al., 2011; Barron et al., 2014; reality testing: Dagnall et al., 2010a; and paranoia: Darwin et al., 2011) and the propensity to validate conspiracist beliefs (Darwin et al., 2011; Swami, 2012; Swami and Furnham, 2012; Brotherton et al., 2013). Schizotypy is a multifactorial psychological construct, covering cognitive, perceptual, and affective domains. Schizotypal traits include suspicion, magical thinking, social anxiety, and paranoia. These factors potentially predispose individuals toward odd and unusual beliefs (Barlow et al., 2009; Darwin et al., 2011). Schizotypy has been defined in different ways, as a milder form of schizophrenia (Rado, 1953; Meehl, 1962), a personality dimension (Eysenck, 1960), and both a healthy variation and a predisposition to psychosis (Claridge, 1997). The latter two perspectives (personality and dual models) are germane to the present paper because they suggest schizotypal traits influence cognitive-perceptual processing within the general population, and contribute to the formation/maintenance of unorthodox/anomalous beliefs (Dagnall et al., 2010b).

Indeed, previous research evinces that individuals scoring higher on positive schizotypy possess stronger belief in anomalous phenomena (generally; Simmonds-Moore, 2010), and the paranormal (specifically; Genovese, 2005; Hergovich and Arendasy, 2007; Hergovich et al., 2008). This summation, however, is overly simplistic and scrutiny reveals a subtle and more sophisticated relationship. There is evidence to suggest that associations between belief in the paranormal and schizotypal factors varies as a function of belief type (Irwin and Green, 1998–1999). Cognitive-perceptual scores are associated with New Age Philosophy, whilst interpersonal deficits relate to belief in extraordinary life forms and witchcraft. Cognitive disorganization influences evaluation of paranormal experiences rather than belief (Irwin, 2009).

These findings extrapolate usefully to the study of conspiracy theories, where studies show a robust relationship between conspiracist ideation and schizotypy (Bruder et al., 2013; Swami et al., 2013; Barron et al., 2014). Pertinently, Darwin et al. (2011) found conspiratorial beliefs correlated positively with paranormal beliefs, paranoid ideation, and schizotypy. Confirmatory analysis revealed schizotypy and paranoid ideation to be important features of conspiratorial thinking. Barron et al. (2014) expanded on these findings by proposing that schizotypal individuals, because of heightened suspiciousness, are more open to arguments in support of conspiracy theories (Darwin et al., 2011). Holm (2009) explained this ‘paranoia-conspiracy link’ in terms of shared, overlapping characteristics. Holm (2009) contends that key tenets of conspiracism (i.e., suspicion and constant fear of external agents) are central characteristics of paranoia.

Darwin et al. (2011) expands upon this idea by proposing that paranoid anxiety may serve an adaptive function. Characteristics associated with paranoid ideation (e.g., low trust, feelings of vulnerability, and belief in the harmful intent of others) may assist the ability to detect potential social threats (Freeman et al., 2005; Gilbert et al., 2005). More pessimistically, the relationship between paranoia and conspiracist thinking may arise from distorted perception and misappreciation of intention/causation (Meller, 2002).

Collectively, studies suggest that believers in conspiracy theories share a propensity to paranoia. However, this supposition is contentious. Whilst, paranoid ideation and conspiratorial beliefs share common features, analysis reveals crucial differences (see Byford, 2011). A key dissimilarity being the non-personal nature of conspiracist ideation, typically paranoid thoughts are self-referenced and focus on the notion of individual threat. Additionally, fear affects conspiracists and individuals suffering from paranoid delusions differently. Conspiracists typically resist and fight, whereas paranoid individuals withdraw. Finally, whilst paranoid delusions are personal, idiosyncratic, and considered implausible, conspiracist beliefs reflect key social events/themes and are often credible.

These differences, together with the observation that conspiracist beliefs are prevalent within the normal population, indicate that explanations attributing conspiracism to negative psychopathology are unsatisfactory. Clearly, conspiracies represent more than just the hallucinations of a deluded, paranoid minority (van der Linden, 2013). A recent study by Wood et al. (2012) may provide a useful framework for contextualizing conspiracism. They found participants endorsed contradictory conspiracy theories. (e.g., the more participants believed Princess Diana faked her own death, the more they believed she was murdered). The authors explained this in terms of coherence with higher-order beliefs (e.g., perceived deception by authority). Adherence to principal beliefs was more important than the congruence of sub-beliefs.

Recent psychological research supports the notion that central, overarching belief systems are important with regard to the acceptance of conspiracy theories (Wood and Douglas, 2013). In this context, the notion of a conspiracist worldview is important. Generally, worldview refers to a set of interrelated assumptions about the nature of the world (Overton, 1991), which act as an interpretive lens for understanding reality and existence (Miller and West, 1993; see Koltko-Rivera, 2004 for detailed review). Specifically, the conspiracist worldview is defined by high-order beliefs (i.e., mistrust of authority, the conviction that nothing is quite as it seems, and deception), which facilitate conspiracist thinking (Goertzel, 1994; Swami et al., 2010; Wood et al., 2012). Thence, the rejection of official accounts is more important than details of individual conspiracy theories.

The worldview concept relates to the notion of conspiracy mentality (Imhoff and Bruder, 2014). Imhoff and Bruder (2014) proposed that endorsement of a specific conspiracy theory largely depends on whether an individual accepts conspiratorial beliefs generally. From this perspective, the conspiracy mentality produces a generalized political attitude associated with the behavioral intention to challenge the status quo and a pejorative view of those in power. In contrast to right-wing authoritarianism and social dominance orientation, conspiracy mentality is related to prejudice against high-power groups that are perceived as less likeable and more threatening than low-power groups. Such traits, however, do not necessarily produce paranoia, and resistance to rational argument. Indeed, when the conspiracy mentality manifests as a desire to seek truth and social advancement it is adaptive.

Given the outlined characteristics of the conspiracist worldview it is likely to incline participants away from analytical-rational processing (reality testing) toward intuitive-experiential processing (Drinkwater et al., 2012). In this context, Pacini and Epstein (1999) and Norris and Epstein (2011) provide a precise delineation of thinking styles. Rational-analytical processing is slow, conscious, considered, and nuanced, whilst intuitive-experiential processing is fast, largely preconscious, holistic, spontaneous, and fairly crude (Irwin and Wilson, 2013).

The latter thinking style would appear to be consistent with the validation of conspiratorial thinking. Indeed, Drinkwater et al. (2012) found reality testing and belief in the paranormal predicted endorsement of General Conspiracist Beliefs (GCB) and specific conspiracies. Proneness to reality deficits and belief in the paranormal were associated with less critical ratings of conspiracy theories (lower truthfulness ratings for official explanations, and positive evaluations of alternative explanations). The tendency toward intuitive-experiential processing may explain recent findings that belief in conspiracy theories was associated with rejection of science (Lewandowsky et al., 2013). Endorsement of conspiracies, therefore results from deference to higher-order beliefs and the failure to appraise thoroughly evidence. Thus, it would appear that elements of conspiracist ideation are adversative to conventional reasoning and scientific thinking.

The notion of a ‘conspiracist worldview’ is demonstrated further by the observation that conspiracism is not limited to specific conspiracy theories, but rather generalizes across conspiracies; individuals who endorse one conspiracy tend to approve others (Goertzel, 1994; Swami et al., 2011; Drinkwater et al., 2012; Lewandowsky et al., 2013). One theory provides evidence for another, reciprocally reinforcing/perpetuating the system (Swami et al., 2011). This effect occurs to the extent that even deliberately deceptive conspiracies (i.e., Red Bull) are endorsed (Swami et al., 2011). Several important papers have viewed these findings as support for Goertzel’s (1994) assertion that conspiracies form part of a monological belief system, a structure that provides easy, automatic explanation for new phenomena, which otherwise would threaten the belief system. However, a recent chapter by Sutton and Douglas (2014) questions this assumption. Particularly, they point out that there are major problems with the monological belief systems position: conspiracy theories are not necessarily mutually supportive, there is a lack of empirical evidence, and alternatives are more plausible. For this reason, the term conspiratorial mindset was preferred.

The present paper examined the notion of conspiracist cognitive style (worldview). Particularly, the authors hypothesized that conspiracist ideation would be associated with higher scores on a range of cognitive-perceptual measures, particularly those tapping into positive schizotypy. To facilitate comparisons with recent published studies schizotypy (Darwin et al., 2011) and delusional ideation (Brotherton et al., 2013) were included. Moreover, the addition of proneness to hallucinations (Launay and Slade, 1981) provided a measure of source monitoring error; assessed the degree to which internally generated imagery is wrongly (misattributed) to an external source (see French et al., 2008). Together, these measures tap into aspects of reality testing; the degree to which, sensory experience is critically assessed (Langdon and Coltheart, 2000) and the plausibility of beliefs evaluated (Irwin, 2004). This hypothesis is consistent with Irwin (2009), who noted that high cognitive-perceptual scores were associated with a preference toward intuitive-experiential thinking. Using a range of measures allowed the researchers to identify, which particular cognitive-perceptual factor(s) were mostly strongly associated with conspiratorial ideation. Previous research has employed these factors independently and thus, it is currently unclear which cognitive-perceptual factor(s) best predict conspiracist ideation.

The present paper employed a range of conspiracist measures because there is no single/accepted measure of conspiracy theory endorsement (Bruder et al., 2013, Sutton and Douglas, 2014). Together, these scales assessed a breadth of conspiracist ideation. This approach had the benefit of facilitating comparisons with previous work, and ensured that findings were not merely an artifact of the measure employed. If conspiracy theories represent a conspiratorial mindset correlations will be evident both between (broad vs. individual) and within (i.e., endorsement of individual conspiracies) conspiracy measures.

## MATERIALS AND METHODS

### PARTICIPANTS

The sample comprised 223 volunteers recruited by researchers in Manchester and the West Midlands using convenience sampling. The mean age was 27.79 years (SD = 12.15) with a range of 18–81 years. Participants included undergraduates, postgraduates, alumni, and employees from the Manchester Metropolitan University.

### COGNITIVE-PERCEPTUAL MEASURES

#### The Schizotypal Personality Questionnaire (SPQ-B; Raine and Benishay, 1995)

The Schizotypal Personality Questionnaire (SPQ-B) is a widely used, easy-to-administer research tool (Bailey and Swallow, 2004), which assesses schizotypal personality disorder, or dimensional schizotypy in non-clinical samples (Jahshan and Sergi, 2007). The SPQ-B is a short version of the 74-item SPQ. The SPQ-B comprises three subscales (cognitive-perceptual, eight items; interpersonal, eight items; and disorganized, six items). Items are presented as statements responded to with “yes” or “no” answers. Yes responses are totalled to produce a score ranging from 0 to 22; upper scores indicate higher levels of self-reported schizotypy. The SPQ-B has established psychometric properties: internal consistency reliability, test–retest reliability, and criterion validity (Raine and Benishay, 1995; Axelrod et al., 2001).

#### The Peters et al. Delusions Inventory (PDI-21; Peters and Garety, 1996; Peters et al., 2004)

The Peters et al. Delusions Inventory-21 measure consists of 21 items with a yes/no response format and assesses delusional ideation in non-clinical populations (Verdoux and van Os, 2002). Particularly, the PDI-21 comprises items measuring: religiosity, persecution, grandiosity, paranormal beliefs, thought disturbances, suspiciousness, paranoid ideation, negative self, depersonalization, catastrophic ideation, and thought broadcast, and ideation of reference and influence (Peters et al., 1999). In addition to the occurrence of delusional ideation, the PDI-21 (using a 5-point scale) also measures level of distress (1 = not at all distressing, 5 = very distressing), preoccupation (1 = hardly ever think about it, 5 = think about it all the time), and conviction (1 = don’t believe it’s true, 5 = believe it is absolutely true). Scores on each dimension range from 0 to 105. Summation of occurrence and dimensional scores produces a composite total (0–336). The scale possesses satisfactory psychometric properties (Jones and Fernyhough, 2007). The PDI-21 has an internal reliability of 0.82, and confirmed test retest reliability (Peters et al., 2004).

#### The Launay–Slade Hallucination Scale (LSHS; Launay and Slade, 1981; Bentall and Slade, 1985)

The Launay–Slade Hallucination Scale (LSHS) assesses inclination to hallucinations in normal individuals (the measure assumes that hallucination experience exists on a continuum with psychological functioning; Slade and Bentall, 1988). The scale contains 12 items, presented as statements. Responses are recorded on a 5-point likert scale, ranging from 0 (certainly does not apply to me) to 4 (certainly applies to me). The LSHS has a total score of 36 (higher scores indicate a greater disposition to hallucination-like experiences). Jones et al. (2009) note that the LSHS satisfactory psychometric properties (internal consistency coefficient = 0.82 (Aleman et al., 2001).

### CONSPIRATORIAL BELIEF MEASURES

#### General Conspiracist Beliefs

Five items measured the degree to which respondents believed that conspiracy theories accurately depict real life events and contain truthful information (e.g., ‘I have heard several conspiracy theories which I believe to be true’; Drinkwater et al., 2012). Responses were measured on a 7-point likert scale (1 = strongly disagree, 4 = neither disagree nor agree, 7 = strongly agree). To control for response bias, the measure contains two reversed items. The GCB has demonstrated internal reliability = 0.72 (Drinkwater et al., 2012).

#### Endorsement of specific conspiracy theories

Ten historical events (e.g., the death of Diana Princess of Wales, the assassination of John Fitzgerald Kennedy) selected randomly from lists of famous conspiracies, assessed endorsement of specific conspiracist beliefs (Vankin and Whalen, 2010; Drinkwater et al., 2012). Two questions followed an outline of each event. Question 1 asked participants to rate the truthfulness of the official explanation (CTO; measured by a 7-point likert scale: 1 = definitely not true, 7 = definitely true), and question 2 asked participants to rate truthfulness of alternative explanations (CTA), the extent to which respondents believed alternative explanations were more truthful than official accounts (measured by a 7-point likert scale: 1 = strongly disagree, 7 = Strongly agree). Scores on the truthfulness sub-scale were reversed in relation to the other conspiracy measures. Hence, high conspiratorial ideation was associated with low endorsement of official explanations. These sub-scales have previously demonstrated adequate internal reliability: CTO = 0.79 and CTA = 0.79 (Drinkwater et al., 2012).

#### Conspiracy Theory Questionnaire (Bruder and Manstead, 2009, see Darwin et al., 2011)

This study used an adapted version of the Conspiracy Theory Questionnaire (CTQ) consisting of 38 items. This measured general belief in conspiracy theory. Participants responded to items (e.g., “there are specialized government services who attempt to harass UFO witnesses into silence”), using an 11-point likert scale (0 = Certainly Not to 100% = Certainly). Possible scores, ranged from 0 to 3800). This measure has excellent internal consistency of 0.96 (Darwin et al., 2011).

### PROCEDURE

Prior to testing ethical approval was granted as part of a wider research project examining the relationship between anomalous beliefs and cognitive-perceptual measures. Potential participants read the information sheet prior to consenting to the study. After providing informed consent, participants received the booklet containing the measures. The instructions asked participants to take their time and answer questions as openly and honestly as possible. The booklet was divided into three sections: personal information (always completed first), cognitive-perceptual measures and conspiracy measures (the order of these latter two sections was counter-balanced across questionnaire booklets).

## RESULTS

### DESCRIPTIVE STATISTICS

Descriptive statistics for cognitive-perceptual and conspiratorial measures appear in **Table 1**. SPQ-B subscale scores (cognitive-perceptual, interpersonal, and disorganized) feature alongside the full-scale score. The PDI-21 produced five scores: item total (yes-no), distress (D), preoccupation (P), conviction (C), and grand total (the summation of the four scores). The final cognitive-perceptual measure, the LSHS, provided an overall score assessing hallucination proneness. Conspiratorial ideation was assessed via a General Conspiratorial Belief (GBC) measure, the CTQ, and endorsement of individual conspiracy theories (evaluated via two scales measuring the degree to which respondents endorsed the official account and the degree to which they endorsed alternative theories). Scale internal reliability was in the acceptable (0.66) to excellent (0.93) range (George and Mallery, 2003).

**Table 1:** Scale descriptive statistics.

Measure	Mean	SD	Minimum–Maximum	α
**Cognitive perceptual measures**
SPQ	6.69	4.38	0–18	0.80
Cognitive-perceptual	2.51	1.97	0–7	0.70
Interpersonal	2.59	2.25	0–8	0.76
Disorganized	1.60	1.59	0–6	0.66
PDI-21 (yes/no)	3.71	3.04	0–16	0.75
Distress	8.37	8.52	0–55	0.78
Preoccupation	9.18	9.22	0–53	0.78
Conviction	11.06	10.53	0–54	0.78
Total PDI	32.33	30.36	0–173	0.93
LSHS	12.29	7.93	0–38	0.82
**Conspiratorial ideation measures**
GCB	3.60	1.14	1.00–6.60	0.80
CTQ	44.13	17.64	4.74–93.42	0.96
Official	4.91	1.12	1.70–7.00	0.85
Alternative	3.27	1.16	1.00–7.00	0.87

SPQ, Schizotypal Personality Questionnaire; PDI-21, Peter’s Delusional Ideation; LSHS, Launay–Slade Hallucination Scale; GCB, general conspiratorial belief; CTQ, Conspiracy Theory Questionnaire; Official, endorsement of official account; Alternative, endorsement of alternative accounts.

#### Conspiratorial measures

Pearson Moment correlations revealed significant correlations between conspiracy measures (GBC, conspiracy theory endorsement, and attitudes towards specific theories) see **Table 2**.

**Table 2:** Conspiracy measure correlations.

	1	2	3	4
(1) GCB				
(2) CTQ	0.56**			
(3) Official	-0.50**	-0.53**		
(4) Alternative	0.59**	0.63**	0.81**	

GCB, general conspiratorial belief; CTQ, Conspiracy Theory Questionnaire; Official, endorsement of official account; Alternative, endorsement of alternative accounts; **p < 0.01.

Within individual conspiracies, all correlations were significant and ranged between 0.50 and 0.81. Individual conspiracy theories also correlated positively (**Table 3**).

**Table 3:** Individual conspiracy theories correlations.

	1	2	3	4	5	6	7	8	9	10	11	12
(1) JFK												
(2) Apollo 11	0.41**											
(3) Elvis	0.23**	0.40**										
(4) Roswell	0.37**	0.47**	0.44**									
(5) Diana	0.47**	0.49**	0.34**	0.37**								
(6) Marilyn	0.41**	0.43**	0.23*	0.41**	0.61**							
(7) Political	0.30**	0.40**	0.23*	0.34**	0.49**	0.41**						
(8) Hitler	0.28**	0.43**	0.37**	0.40**	0.47**	0.50**	0.39**					
(9) Climate	0.29**	0.38**	0.41**	0.40**	0.37**	0.36**	0.43**	0.39**				
(10) WTC	0.40**	0.51**	0.42**	0.36**	0.50**	0.42**	0.47**	0.45**	0.42**			
(11) GCB	0.39**	0.48**	0.37**	0.40**	0.47**	0.36**	0.37**	0.43**	0.29**	0.45**		
(12) CTQ	0.43**	0.40**	0.24**	0.40**	0.55**	0.45**	0.50**	0.41**	0.33**	0.54**	0.56**	

JFK, Kennedy assignation; Apollo 11, moon landing; Elvis, death of Elvis Presley; Roswell, Roswell incident; Diana, Death of Diana Princess of Wales; Marilyn, Death of Marilyn Munroe; Political, political manipulation of the masses; Hitler, Death of Adolf Hitler; Climate, global warming; WTC, World Trade Centre attack; GCB, General Conspiracist Beliefs; CTQ, Conspiracy Theory Questionnaire. **p < 0.01.

#### Cognitive-perceptual and conspiratorial measure correlations

Pearson Product Moment correlations examined relationships between cognitive-perceptual and conspiratorial ideation measures (see **Table 4**). Cognitive-perceptual measures (SPQ-B, PDI-21, and LSHS) correlated significantly across conspiratorial measures. The cognitive-perceptual SPQ subscale produced significant correlations, whilst the interpersonal and disorganized factors produced weaker, inconsistent results.

**Table 4:** Correlations conspiracy measures and cognitive-perceptual measures.

	CP	INT	DIS	SPQ	PDI-21	*D*	*P*	*C*	Total PDI	LSHS
GCB	0.33**	0.11	0.12**	0.25**	0.27**	0.27**	0.27**	0.26**	0.28**	0.19**
CTQ	0.36**	0.18**	0.18**	0.32**	0.44**	0.38**	0.43**	0.45**	0.44**	0.31**
Official	-0.26**	-0.05	-0.10	-0.18**	-0.33**	-0.31**	-0.35**	-0.37**	-0.36**	-0.13*
Alternative	0.35**	0.10	0.19**	0.28**	0.42**	0.43**	0.45**	0.45**	0.45**	0.17**

CP, cognitive-perceptual; INT, interpersonal; SPQ, Schizotypal Personality Questionnaire; PDI-21, Peter’s Delusional Ideation; D, Distress; P, Preoccupation; C, Conviction; Total PDI, PDI grand total; LSHS, Launay–Slade Hallucination Scale; GCB, general conspiratorial belief; CTQ = Conspiracy Theory Questionnaire; Official, official account endorsement; Alternative, endorse alternative theories. *p < 0.05; **p < 0.01.

#### Canonical correlation analysis

Canonical analysis assessed the relationship between cognitive-perceptual variables (SPQ factors, overall PDI, and LSHS) and conspiracy measures. Canonical analysis is a multivariate technique that focusses on measuring correlations between variable clusters. Assessing cluster relationships, rather than individual correlation coefficients, reduces the likelihood of Type I error (Tabachnick and Fidell, 1996). The aim of canonical analysis is to derive a linear combination (synthetic variable) from each variable set (i.e., cognitive-perceptual and conspiracy measures), which can be used to discern relationships between multiple predictor and criterion variables.

Cognitive-perceptual measures were entered as predictors of conspiracy measures. The analysis produced four functions with squared canonical correlations (*R_c_^2^*) of 0.26, 0.04, 0.02, and 0.01 for each, consecutive function. Across functions the full model was statistically significant, Wilks’s λ = 0.67, *F*(20,707.39) = 4.38, *p* < 0.001. Wilks’s λ represents the unexplained variance of the overall model, and 1–λ provides the effect size total model. Using this criterion, the effect size for the four canonical functions was 0.32, indicating the model accounted for 32% of the shared variance between the variable sets (a medium effect size).

Dimension reduction analysis tested the hierarchal arrangement of functions. Only the first function explaining 26.1% of variance in the data was significant. The standardized canonical function coefficients and structure coefficients for Function 1 (cognitive-perceptual facets of conspiracist ideation) appear in **Table 5**. Functions 2–4, 3–4, and 4 were not statistically significant, *F*(12,566.48) = 1.54, *p* > 0.05; *F*(6,430.0) = 1.31, *p* > 0.05; and *F*(2,216.0) = 1.06, *p* > 0.05, respectively. Primary contributors to the predictor synthetic variable (conspiracy measures) were SPQ cognitive-perceptual and delusional ideation scores, LSHS provided a secondary contribution. All criterion variables made primary contributions to the synthetic criterion variable, possessing structure coefficients greater than 0.45. The squared structure coefficients supported this conclusion.

**Table 5:** Canonical solution for cognitive-perceptual variables as predictors of conspiracy measures for function 1 (cognitive-perce ptual facets of conspiracist ideation).

	Function 1		
Variable	Coefficient	*r_s_*	$r_{s}^{2}$*(%)*
**Conspiratorial ideation**
GCB	-0.02	0.61	37.21
CTQ	0.50	0.87	75.69
Official	0.12	-0.71	50.41
Alternative	0.72	0.92	86.64
*R^2^*			26.10
**Cognitive-perceptual**
SPQ			
Cognitive-perceptual	0.30	0.76	57.76
Interpersonal	-0.05	0.30	9.00
Disorganized	0.07	0.42	17.64
PDI	0.81	0.96	92.16
LHSH	-0.07	0.58	33.64

Structure coefficients (*r_s_*) greater than 0.45 are underlined. Coefficient*,* standardized canonical function coefficient; *r_s_*, structure coefficient; $r_{s}^{2}$, squared structure coefficient.

#### Conspiracism and cognitive-perceptual factors

For completeness, partial correlations assessed the relative contributions of schizotypy and delusional ideation. A significant positive correlation was observed between PDI-21 and CTQ, controlling for SPQ, *r* = 0.33, df = 219, *p* < 0.001. Contrastingly, the correlation between SPQ and CTQ, controlling for PDI-21, *r* = 0.10, df = 219, *p* > 0.05 was not significant. This indicated that the relationship between schizotypy and conspiracist beliefs was principally attributable to PDI.

## DISCUSSION

As hypothesized, conspiracist thinking was associated with positive schizotypy. The cognitive-perceptual subscale of the SPQ-B (Raine and Benishay, 1995) correlated consistently across conspiracy measures, whilst interpersonal, and disorganized factors produced weaker, inconsistent relationships. Considering the influence of individual measures, delusional ideation was most strongly associated with conspiracism. Indeed, schizotypy failed to add unique variance. This finding indicated that the correlation between schizotypy and conspiracism was largely attributable to the SPQs indirect measurement of delusional ideation.

In the context of the present study, it is worth noting that paranoia is a central theme within the PDI-21 (Peters and Garety, 1996; Peters et al., 2004); the scale contains multiple items measuring persecution, suspiciousness, and paranoid ideation. Thus, generally, findings concur with Darwin et al. (2011), who reported a link between paranoid/delusional/ideation and belief in conspiracies (Jones and Fernyhough, 2007). However, the notion that schizotypal individuals are more open to arguments in support of conspiracy theories as a result of heightened suspiciousness, (Darwin et al., 2011; Barron et al., 2014) is contentious. Suspiciousness *per se* may bias individuals away from externally generated arguments toward internally generated or self-affirmed views of the world but will not necessarily produce conspiratorial thinking. In this context, conspiracy theories may confirm rather than shape conspiracist notions.

Clearly, delusional ideation, and belief in conspiracies share important cognitive characteristics (i.e., unusual beliefs, magical thinking, fear of external agencies and persecutions). This is evident when typical features of conspiratorial thinking (worldview) are considered. Particularly, the conviction that unorthodox theories/explanations are true, in the face of overwhelming contradictory evidence, and the presumption of deception are prominent features of conspiracist thinking (e.g., Sunstein and Vermeule, 2009). The PDI-21, however, assesses only certain types of sub-clinical delusions (Jones and Fernyhough, 2007). Hence, future studies may wish to investigate which explicit elements (beyond paranoia) of delusional thinking contribute to the development and maintenance of conspiratorial thinking. Moreover, the relationship between paranoia and conspiratorial thinking requires further explication. For example, recent work by Prooijen and Dijk (2014) found that inducing empathy increased collective paranoia in the form of conspiracy beliefs.

Whilst, the reporting of an association between conspiracism and paranoid/delusional ideation is consistent with previous research (e.g., Holm, 2009; Darwin et al., 2011), further consideration is required. Cognitive-perceptual factors in combination accounted for only 32% of the variance. Thus, within normal populations, other variables must also influence the development and maintenance of belief in conspiracies (the conspiracist worldview). This is perhaps, not surprising when the development of the PDI-21 is considered. The measure assesses tendency to delusional ideation in non-clinical populations and samples as wide a variety of delusions as possible (Jones and Fernyhough, 2007). High PDI-21 item endorsement rates within the general population indicate only that delusional themes are present within conspiractorial ideation and supports the notion that normal and deluded samples overlap.

Within the present study, strong associations were evident between conspiracy measures. Not only did general attitudes to conspiracy theories (GBC) correlate with scores on the CTQ, and endorsement of specific conspiracy theories, but also individual conspiracy theory ratings correlated. These results are supportive of a conspiratorial mindset; individuals who endorse one conspiracy are likely to validate others (Goertzel, 1994; Swami et al., 2011; Drinkwater et al., 2012). One conspiracy provides evidence for another and beliefs are reciprocally reinforcing (Swami et al., 2011). The conspiratorial mindset, provides a framework for accommodating new phenomena, which otherwise would threaten the belief system (Goertzel, 1994). From this perspective, conspiracism provides individuals with a sense of ontological security, order and continuity in life experiences (Giddens, 1991). This finding accords with preceding research and is indicative of a conspiratorial worldview.

Cognitions arising from this system may influence reasoning and decision-making. A preference for intuitive-experiential processing (Drinkwater et al., 2012) and an adherence to high-order beliefs (Wood et al., 2012) is likely to result in a thinking style (worldview), which is internally coherent, but subjectively flawed in relation to external evidence. Whilst cognitions, in the context of conspiracies, are rational (in a bounded sense) and consistent from the individual’s perspective, they are likely to produce traits resembling the positive symptoms of schizotypy (i.e., ideas of reference, odd beliefs, magical thinking, and paranoid ideation). Unless cognitions are unduly disruptive (high schizotypy), the resultant schizotypal symptoms should be viewed as benign/healthy (Holt et al., 2008). This may explain why some researchers view traits, such as paranoid anxiety as adaptive (Freeman et al., 2005).

Clearly, more work is required in this area. Conspiratorial thinking is complex and more than the mere product of poor psychopathological functioning. Indeed, recent work views conspiracist ideation as a subset of false beliefs that help individual’s comprehend phenomena outside of their control (Swami and Furnham, 2014). Even this delineation is problematic because it assumes wrongly that conspiratorial beliefs are necessarily false, this may not always be the case. The key point is that endorsement of non-conventional theories/notions arises from limited consideration, or selective interpretation of available evidence. Likewise, accepting prevailing accounts without deliberation may represent illogical reasoning.

In this context, it is important to note that thinking styles are not exclusive, both intuitive-experiential and analytical-rational thought contribute to behavior (Pacini and Epstein, 1999). Although, thinking style varies in part with context, people may have preferences for using one style more routinely. An inclination towards intuitive-experiential thinking, within the normal population, is likely to produce domain specific truncated reasoning and irrational beliefs (results in a conspiracist worldview).

Conspiracism is common within modern society and despite criticism, prevails within the normal population. For these reasons, more research is required. One potential avenue, similar to work in the area of paranormal belief, may be to examine whether conspiracism is associated with particular probabilistic reasoning errors/bias. For example, Leman and Cinnirella (2007) suggest that people generally believe that significant events require major causes. This heuristic may explain why important social happenings are often the focus of conspiratorial thinking. Indeed, recent work proposes an association between conspiratorial belief and domain-general susceptibility to conjunction fallacy (see Brotherton and French, 2014). However, because few papers exist further work is necessary. This area may thus be a fertile and fruitful area of academic endeavor.

In summary, this paper has produced several important findings. Whilst delusional ideation is associated with, conspiratorial thinking in the sub-clinical population, delusional ideation is not a major determining factor. Indeed, collectively cognitive-perceptual factors explained only a relatively small proportion of the variance in conspiracist ideation. Thus, other variables, such as preferential thinking style are also likely to influence the inclination to endorse conspiracist beliefs. In this context, the present paper suggests that previous research has overemphasized the role of paranoia. Typically, studies argue that conspiracy theories arise from a propensity to paranoia. However, the characteristics of paranoia differ in critical ways from typical paranoid ideation (see Byford, 2011); they are non-personal, self-referenced, and focus on the notion of individual threat. Hence, conspiracists typically resist and fight and their beliefs (often credibly) reflect key social events/themes. Consequently, the conspiracy mentality is often adaptive and hence manifests in a need to seek truth and social advancement. This positive view of conspiratorial thinking runs contrary to the typical pejorative interpretation of conspiracist ideation.

## Conflict of Interest Statement

The authors declare that the research was conducted in the absence of any commercial or financial relationships that could be construed as a potential conflict of interest.
